# Characterization of *Naegleria fowleri* from two human cases: insights into its excretion/secretion products

**DOI:** 10.3389/fcimb.2025.1585448

**Published:** 2025-05-06

**Authors:** Natalia Chacón Camacho, María Fernanda Steller Espinoza, Johan Alvarado-Ocampo, Antonio Osuna, Lissette Retana Moreira, Elizabeth Abrahams Sandí

**Affiliations:** ^1^ Departamento de Parasitología, Facultad de Microbiología, Universidad de Costa Rica, San José, Costa Rica; ^2^ Centro de Investigación en Enfermedades Tropicales (CIET), Universidad de Costa Rica, San José, Costa Rica; ^3^ Grupo de Bioquímica y Parasitología Molecular (CTS 183), Departamento de Parasitología, Instituto de Biotecnología, Universidad de Granada, Granada, Spain

**Keywords:** *Naegleria fowleri*, trophozoites, excretion/secretion products, protein, cytotoxicity

## Abstract

**Introduction:**

*Naegleria fowleri* is the etiologic agent of primary acute meningoencephalitis (PAM). Although this amoeba is commonly found in water and soil, reports of infections are rare; problems with diagnosis probably contribute to underestimation. Moreover, information regarding the pathogenicity of this species is still lacking. Costa Rica reported the first three cases of PAM during 2020; from these, two *N. fowleri* isolates were recovered. The aim of this study was to characterize and compare these isolates, focusing in excretion/secretion products.

**Methods:**

Analyses of protein profiles by silver staining and protease activity assays were performed to characterize whole protein extracts and conditioned media from isolates. Proteomic analyses of excretion/secretion products, including extracellular vesicles (EVs), were performed, and cytopathic effect and drug susceptibility tests were also compared between isolates.

**Results:**

Results obtained were similar for both isolates. Patterns of multiple bands were observed in each isolate after silver staining. Proteomics analyses revealed a total of 88 and 62 non-redundant proteins as part of the cargo of EVs secreted by trophozoites of *N. fowleri* Guanacaste and *N. fowleri* Limón, while conditioned media results revealed 34 and 17 non-redundant proteins, respectively; hydrolase activity and actin filament binding were part of the most represented gene ontology terms in EVs and conditioned media. Regarding drug susceptibility assays, no statistically significant differences were identified. On the other hand, although protease activity resulted very similar with substrate buffer at pH 8.5, only *N. fowleri* Limón showed activity between 40 and 70 kDa at pH 5.0; in contrast, a more rapid cytopathic effect was observed when incubating Vero cells with *N. fowleri* Guanacaste.

**Discussion:**

Comparative analyses of different *N. fowleri* isolates, supported by their remarkable genomic heterogeneity that could be reflected in different metabolic repertoires, are key to understanding virulence and pathogenicity of this amoeba, and could help to explain whether different isolates differ in the severity or course of PAM.

## 1 Introduction

*Naegleria fowleri* is a thermophilic free-living amoeba (FLA) of worldwide distribution that can be found mainly in freshwater and soil (Visvesvara and Stehr-Green, 1990). This species is the etiologic agent of primary acute meningoencephalitis (PAM), a central nervous system (CNS) infection of acute and fulminant course, with initial symptoms indistinguishable from bacterial meningitis (Retana Moreira et al., 2024), which complicates its diagnosis. The infection usually occurs when water with the amoebae enters the nose and gains access to the brain, migrating along the olfactory nerves through the cribriform plate (Cope et al., 2015). The incubation period varies from two to fifteen days and death typically occurs from three to seven days after the onset of symptoms (Yoder et al., 2010).

Since the first description of PAM (Fowler and Carter, 1965), more than 445 cases have been reported, reaching fatality rates of 97% (Cope et al., 2015). In this sense, most cases have been described in the United States, which accounts for more than one third; reports of PAM have also been published in other countries like Bangladesh, Pakistan and Australia, among others (Cope et al., 2015).

Regarding Latin America, PAM cases have occurred in Mexico, Cuba, Puerto Rico, Brasil, Colombia, Perú, Argentina, Venezuela and Costa Rica (Cabello-Vílchez, 2023), country in which three cases were diagnosed during the first trimester of 2020 (Retana Moreira et al., 2020); of these cases, one patient successfully recovered from the infection after the treatment (Retana Moreira et al., 2020). Prior to the diagnosis of these cases, an environmental investigation in the country confirmed the presence of *N. fowleri* in water sources at a resort that was visited by a child from the United States who died of PAM after returning to his country, in 2014 (Abrahams Sandí et al., 2015).

The diagnostic confirmation of PAM cases in Costa Rica allowed the isolation and axenic culture of trophozoites of *N. fowleri* from clinical samples (cerebrospinal fluid) of two of the three infected patients. These *N. fowleri* isolates have allowed research in extracellular vesicles (EVs) performed by our group to date (Retana Moreira et al., 2022, 2024); however, comparisons between trophozoites and conditioned media of these two isolates have not been performed yet. As an example, in 2007, Serrano Luna et al. (2007) characterized *N. fowleri* strains isolated from two human cases of PAM in México by conventional and cellular and molecular techniques, revealing no differences in the protein patterns and proteases of the strains (Serrano Luna et al., 2007). However, in a more recent study, Russell and Kyle (2022) found significant differences in the susceptibilities of different clinical isolates to seven of eight drugs tested, as well as significant variances in growth rates among the isolates (Russell and Kyle, 2022).

The aim of this study is to characterize and compare two autochthonous isolates of *N. fowleri* from Costa Rica employing several approaches, which include analyses of protein profiles, protease activity and cytopathic effect, as well as proteomics analyses of excretion/secretion (E/S) products. Drug susceptibility tests are also included.

## 2 Materials and methods

### 2.1 Axenic culture of *Naegleria fowleri* trophozoites

Trophozoites of two clinical isolates of *Naegleria fowleri* (*N. fowleri* Guanacaste and *N. fowleri* Limón; accession numbers: MT090627 and MT210902) were cultured in 75 cm^2^ flasks (Cellstar, Greiner Bio-One, Austria) with 2% casein hydrolysate (Sigma Aldrich, USA) culture medium, supplemented with 10% inactivated fetal bovine serum (FBS) (Gibco, USA) and antibiotics (penicillin/streptomycin). The flasks were incubated at 37°C, with daily observation of the amoebae under an inverted microscope.

### 2.2 Cell line

Vero cells (ATCC CCL-81) were cultured in 75 cm^2^ flasks (Corning lncorporated, USA) with RPMI culture medium (Gibco, USA) supplemented with 10% FBS and antibiotics. Cells were subcultured as described by Ammerman et al. (2008) and maintained at 37°C and 5% CO_2_.

### 2.3 Preparation of whole protein extracts of *N. fowleri* trophozoites and conditioned media

Trophozoites of each *N. fowleri* isolate (5 x 10^7^) were obtained from the culture flasks as previously described by Rizo-Liendo et al. (2019). Amoebae were washed three times in sterile PBS by centrifugation at 2,000 rpm for five minutes and the pellet was resuspended in 500 µL PBS. Whole protein extracts of amoebae were obtained after three sonication cycles (20 seconds, with one minute pause between cycles) and an output of 25, in a 4710 Series Ultrasonic Homogenizer (Cole Parmer, USA). The samples were then aliquoted and stored at -80°C until use.

Conditioned media of each *N. fowleri* isolate were obtained after the replacement of culture medium with 2% casein hydrolysate (without fetal bovine serum nor antibiotics) and incubation at 37°C for five hours. After this time, the media was collected and centrifuged at 3,500 rpm for 15 minutes at 4°C and the supernatant obtained was filtered through 0.22 µm pore filters (Sartorius, Germany). Protein quantification of the samples was performed using the MicroBCA™ protein assay kit (Thermo Fisher Scientific, USA), following the manufacturer’s instructions.

### 2.4 Protein profiles of whole protein extracts of *N. fowleri* trophozoites

Whole protein extracts of *N. fowleri* trophozoites (undiluted protein amount: 10 µg) were subjected to electrophoresis in 12% SDS-polyacrylamide gels. The electrophoresis was run at 100 V for approximately one hour in a Mini-Protean 3 Cell electrophoresis chamber (Bio-Rad Laboratories Inc, USA) and the gels were stained using silver stainings, as described elsewhere (Heukeshoven and Dernick, 1988).

### 2.5 Proteases determination in whole protein extracts of *N. fowleri* trophozoites and conditioned media by zymography

The evaluation of the presence and type of proteases in whole protein extracts and conditioned media of each *N. fowleri* isolate was performed using 12% SDS-polyacrylamide gels with 1% gelatin as the protease substrate, according to the protocol of Herron et al. (1986), with runs performed at 180 V, on ice. Prior to the sample loading onto the gels (protein amount: 30 ug), these were incubated with different protease inhibitors for one hour at 37°C: 5 µM iodoacetamide (Sigma-Aldrich, USA) as cysteine proteases inhibitor, 5 mM phenylmethylsulfonyl fluoride (PMSF) (Sigma-Aldrich, USA) as serine proteases inhibitor and 2 mM ethylenediaminetetraacetic acid (EDTA) (Sigma-Aldrich, USA) as metalloproteases inhibitor (Vyas et al., 2015; Lam et al., 2017; Retana Moreira et al., 2020).

The gels were then washed in 1% Triton X-100 for 30 minutes and incubated overnight at 37°C in the substrate buffer (Retana Moreira et al., 2020). In this case, three substrate buffers with different pH were tested: i) acetic acid 100 mM/2 mM CaCl_2_ (pH 3.0), ii) 100 mM sodium acetate/2 mM CaCl_2_ (pH 5.0) and iii) Tris 50mM HCl/5mM CaCl_2_ (pH 8.5). After the incubations, the gels were stained with Coomassie R-250 at 0.2% in fixative solution (40% methanol and 10% acetic acid) for one hour, for further destaining to observe protease digestion bands according to the method of Hadas and Mazur (1993).

### 2.6 Proteomics analyses of *N. fowleri* excretion/secretion products

Protein identification in conditioned media of each *N. fowleri* isolate was performed using proteomics. For this purpose, samples were submitted to electrophoresis and the electrophoretic runs were stopped as soon as the migration front entered 3 mm into the resolving gel, so that the whole protein cargo was concentrated as a single band in the stacking/resolving gel interface, as previously performed (Retana Moreira et al., 2020). The bands were visualized after Coomassie blue staining and then excised from the gels, destained in 50% acetonitrile, and submitted to tryptic digestion and nano-LC-MS/MS analysis at the “Instituto Clodomiro Picado”. The MS/MS spectra obtained were processed for comparison and pairing of the peptides with protein sequences from *Naegleria* sp. contained in the database UniProt, using Peaks X^®^ (Bioinformatics Solutions, Waterloo, ON, Canada).

Proteomic analyses of extracellular vesicles secreted by each *N. fowleri* isolate were also achieved in this work. For this purpose, EVs secreted by trophozoites were isolated and characterized following previously described protocols (Retana Moreira et al., 2022, 2024), in accordance to the Minimal Information for the Study of Extracellular Vesicles (MISEV) guidelines (Welsh et al., 2024). Briefly, 5 × 10^7^ trophozoites were incubated for five hours at 37°C in 75 cm^2^ Nunc EasYFlask cell culture flasks (Thermo Fisher Scientific, USA) with 3.5 mL of 2% casein hydrolysate (Sigma Aldrich, US) culture medium without serum nor antibiotics. After this incubation, the supernatants were collected and submitted to differential centrifugation steps (3,500 × g for 15 minutes at 4°C and 16,000 × g for 30 minutes at 4°C), filtration using 0.22 μm pore filters (Sartorius, Germany) and final ultracentrifugation steps at 120,000 × g for 150 minutes at 4°C (Retana Moreira et al., 2024). The resulting pellets were washed two times in sterile filtered (0.22 μm pore filter) PBS at 120,000 × g for 150 minutes and suspended in 100 μL sterile PBS. Characterization analyses, including electron microscopy, were performed, and protein quantification was achieved using microBCA™ (Thermo Fisher Scientific, USA). Mean size of EVs obtained were within the range of 216 nm - 268 nm using nanoparticle tracking analysis.

Electrophoresis and proteomic analyses of EVs secreted by trophozoites of each *N. fowleri* isolate were performed as described above for conditioned media.

### 2.7 Evaluation of the cytopathic effect of *N. fowleri* trophozoites and conditioned media

To evaluate the cytopathic effect of trophozoites and conditioned media of each *N. fowleri* isolate, two different approaches were employed: i) crystal violet staining and ii) determination of lactic dehydrogenase (LDH) release. In both cases, ratios of 1 amoeba per 2 cells were employed.

#### 2.7.1 Crystal violet staining

Vero cells were seeded on 24-well plates (Corning Costar, USA) until confluence. Then, cells were infected using a MOI of one amoeba per two cells, for further incubation for five hours in 2% casein hydrolysate culture medium. Cell destruction by the amoebas was qualitatively determined by crystal violet staining, employing the methodology of Khan et al. (2000). Briefly, 200 µL of 10% formalin (Sigma-Aldrich, USA) were added to each of the wells and incubated for 30 minutes at room temperature. Then, the content was removed (medium + formalin) and 200 µL of crystal violet were added to each well and incubated for 45 minutes at room temperature. Finally, the dye was removed by inversion of the plate, allowed to dry and placed in a light chamber to observe the detachment of the Vero cell monolayer.

#### 2.7.2 Quantification of lactate dehydrogenase release

The cytotoxicity of trophozoites and conditioned media of each *N. fowleri* isolate over Vero cells was evaluated using the Pierce LDH cytotoxicity assay kit (Fisher Scientific, USA), following the manufacturer’s indications. Briefly, 10 µL of each of the following solutions were added to a 96-well plate (Greiner Bio One Cellstar, Austria) with a confluent monolayer of Vero cells: 1.25 x 10^5^ trophozoites, conditioned medium (protein concentration: 5 µg/µL), water (control of spontaneous activity) and lysis buffer (control for maximum LDH activity). The plate was incubated at 37°C for five hours and the enzyme activity was monitored every hour. In this sense, after each hour of incubation, 50 µL of supernatants from the corresponding wells were transferred to another empty 96-well plate and 50 µL of the reaction mixture of the kit were added, for further incubation for 30 minutes at room temperature. Finally, 50 µL of the stop solution was added to all wells and the absorbance was measured at 490 nm and 680 nm in a microplate reader (Synergy HT, BioTek, USA).

### 2.8 Effect of *N. fowleri* conditioned media and extracellular vesicles over cell viability

The effect of conditioned media and EVs over cell viability was evaluated using Presto Blue™ cell viability reagent (Thermo Fisher Scientific, USA). In this case, 90 µL of conditioned media (protein concentration: 16.0 µg/µL) or EVs (protein concentration: 1.6 µg/µL) were added to Vero cells seeded in a 96-well plate (Greiner Bio One Cellstar, Austria) and, after a five-hour incubation at 37°C, 10 µL of PrestoBlue™ reagent were added. The plate was incubated for 24 hours at 37°C and then, the absorbance of the plate was determined at a wavelength of 570 nm excitation and 600 nm emission in a microplate reader (Synergy HT, BioTek, USA). Cells in culture media (RPMI and RPMI supplemented with FBS and antibiotics) and cells treated with 1% triton X-100 were employed as viability and death controls, respectively.

### 2.9 Drug susceptibility assays

The effect of eight concentrations of miltefosine (initial concentration: 320 µg/mL), voriconazole and amphotericin B (initial concentrations: 250 µg/mL) were tested on each *N. fowleri* isolate. For these experiments, 50 µL of the trophozoites of both isolates (10^5^ amoebae) were placed in 96-well plates (Greiner Bio-One Cellstar, Austria). After trophozoites adhered to the plate, the supernatant was carefully removed and 90 µL of the respective dilutions of the drugs and 10 µL of the Presto Blue™ reagent were added. The plates were incubated for 72 hours at 37°C and, after this time, spectrophotometric reading was performed at an excitation wavelength of 570 nm and 600 nm emission, in a microplate reader (Synergy HT, BioTek, USA).

To estimate the cytotoxic concentration 50 (CC50), 50% of the value of the absorbance of growth control was determined. Then, the concentration in which the absorbance value was the closest below that value of 50% of the growth control was identified as the concentration that caused the inhibition of 50% of the amoebas.

### 2.10 Statistical analyses

Comparative qualitative analyses were carried out for electrophoresis, zymographies and determination of cytopathic effect using crystal violet staining.

For LDH assays, viability tests using PrestoBlue™ reagent and drug susceptibility assays, one-way analysis of variance (ANOVA) tests were performed, with *post-hoc* comparisons using Tukey test. The differences were considered significant with *p* < 0.05 and analyses were performed in R.

Each assay was performed by triplicate.

## 3 Results

### 3.1 Protein profiles of whole protein extracts of *N. fowleri* trophozoites

**Figure 1:**
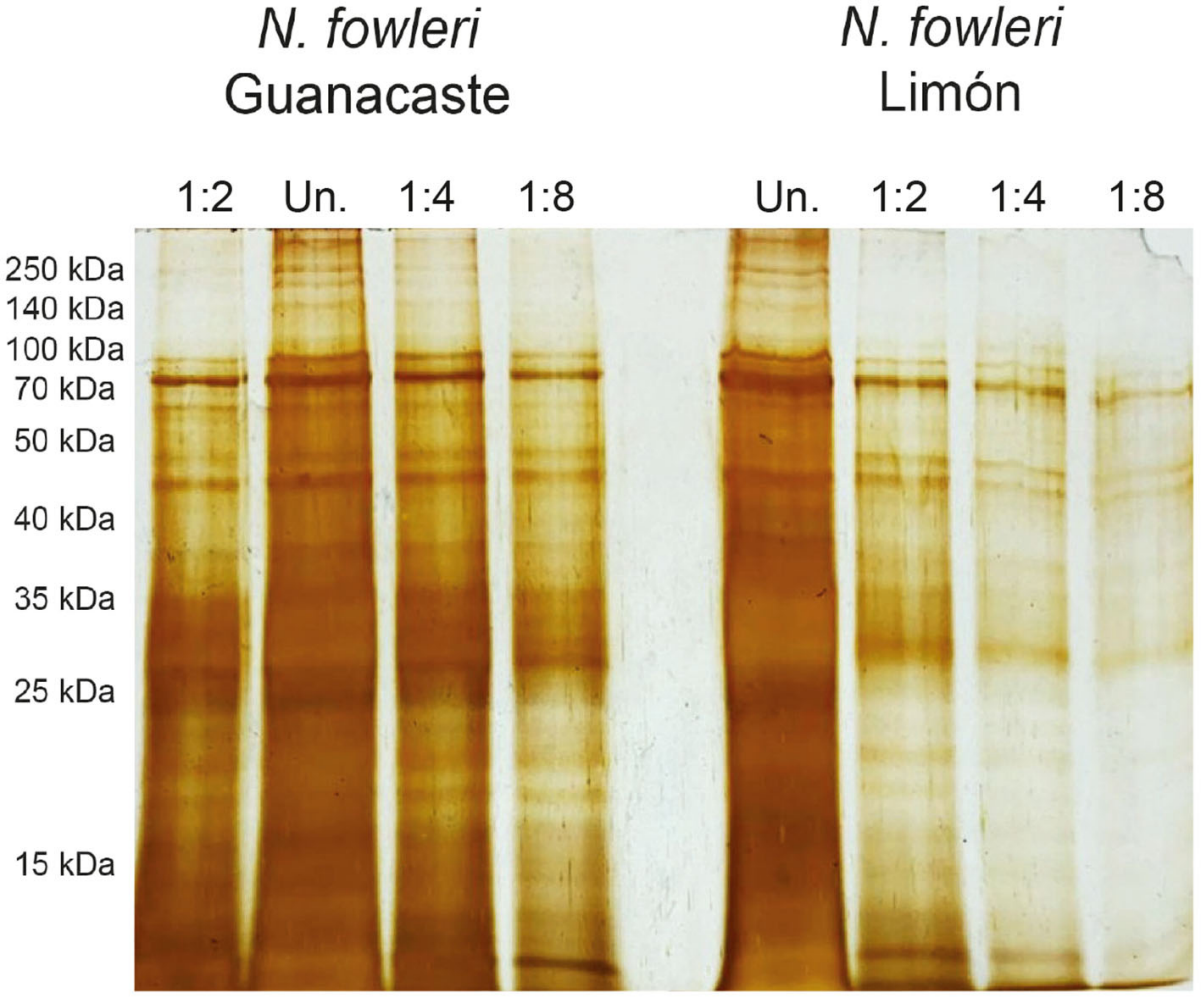
Protein profiles of whole protein extracts of trophozoites of *Naegleria fowleri* Guanacaste and *N. fowleri* Limón after SDS-PAGE electrophoresis and silver staining. Results revealed the presence of bands between 10 and 250 kDa, with an area of multiple bands below 35 kDa. Protein concentration of undiluted samples (Un.) was 1.0 µg/µL and the total amount of protein loaded onto the gels was 10 µg.

Protein profiles of the axenic *N. fowleri* isolates were analyzed after SDS-PAGE electrophoresis and silver staining (**Figure 1**). As shown in **Figure 1**, silver staining revealed the presence of protein bands between 10 and 250 kDa, with an area of multiple bands below 35 kDa. Using this staining, four well-defined protein bands between approximately 45 and 60 kDa were observed, in addition to a very evident band of approximately 10 kDa.

### 3.2 Proteases determination in whole protein extracts of trophozoites and conditioned media

**Figure 2:**
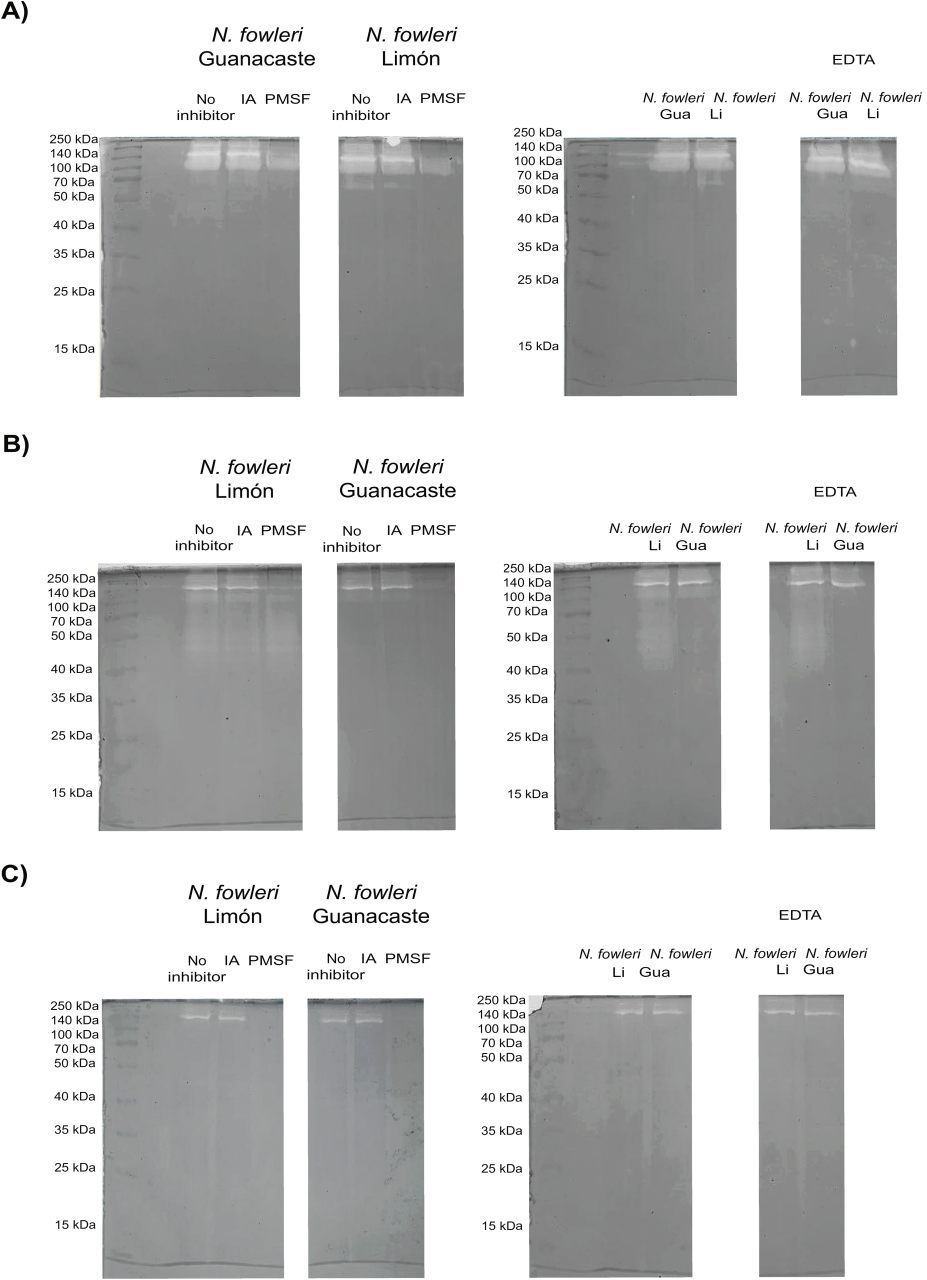
Zymographies that show gelatin digestion bands by whole protein extracts of trophozoites of *Naegleria fowleri* Guanacaste and *N. fowleri* Limón. Protease activity after the incubation of samples with different protease inhibitors was evaluated; for these purposes, iodoacetamide (IA), pheylmethylsulphoyl fluoride (PMSF) and ethylenediamineteraacetic acid (EDTA) were employed as cysteine, serine and metalloproteases inhibitors, respectively. Protease activity was evaluated at different pHs: **(A)** pH 8.5 (50 mM Tris-HCl/5 mM CaCl_2_); **(B)** pH 5.0 (100 mM sodium acetate/2 mM CaCl_2_); and **(C)** pH 3.0 (100 mM acetic acid/2 mM CaCl_2_). The highest protease activity was observed when performing the incubations at pH 8.5. Protein concentration of the samples was 5.0 µg/µL. Gua, Guanacaste; Li, Limón.

**Figure 3:**
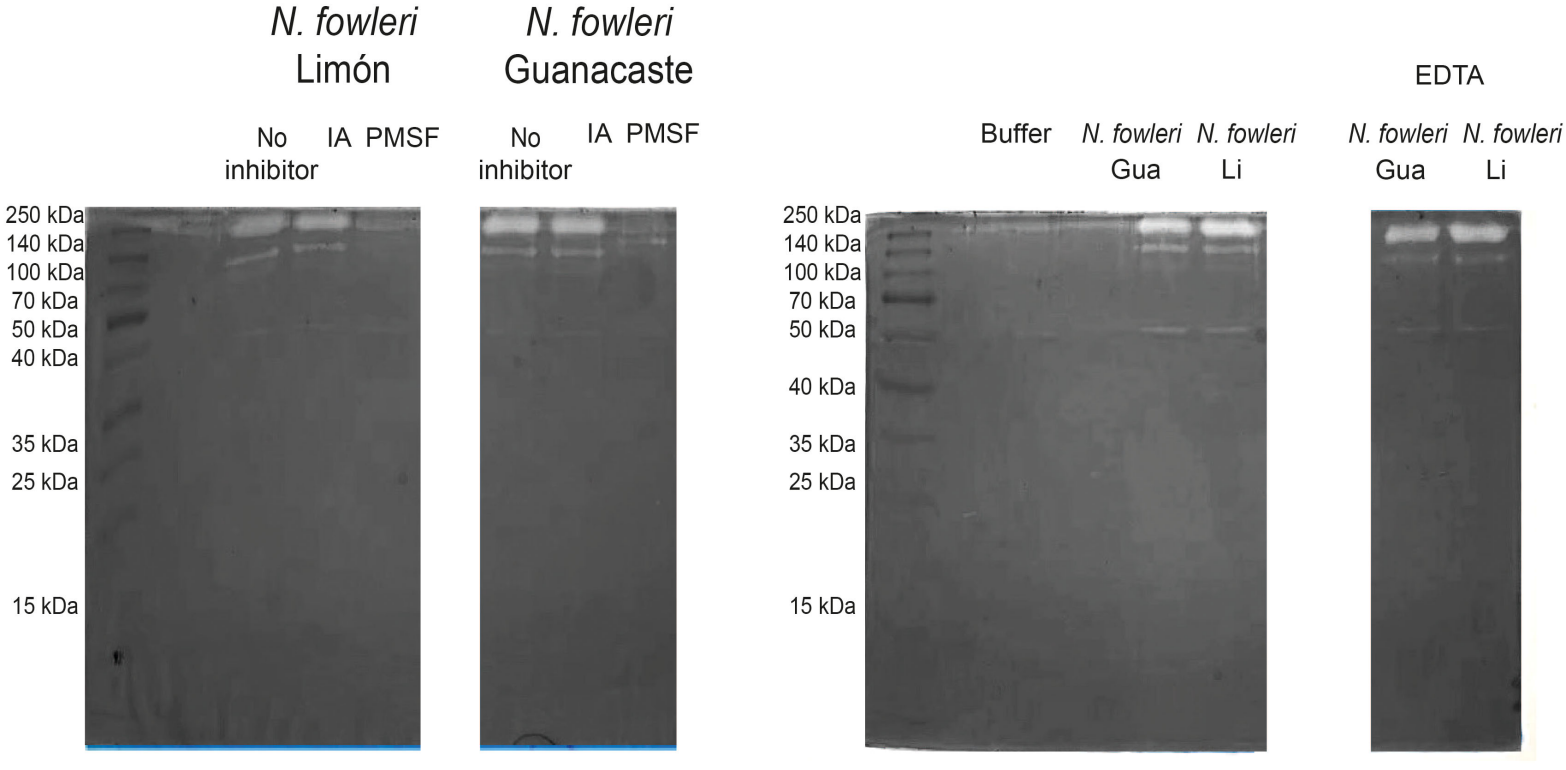
Zymographies that show gelatin digestion bands by conditioned media of *Naegleria fowleri* Guanacaste and *N. fowleri* Limón. Protease activity after the incubation of samples with different protease inhibitors was evaluated; for this purpose, iodoacetamide (IA), phenylmethylsulphonyl fluoride (PMSF) and ethylenediaminetetraacetic acid (EDTA) were employed as cysteine, serine and metalloproteases inhibitors, respectively. A substrate buffer at pH 8.5 (50 mM Tris-HCl/5 mM CaCl2) was employed. Protein concentration of the samples was 5.0 µg/µL. Gua, Guanacaste; Li, Limón.

Protease activity of whole protein extracts and conditioned media obtained from the axenic cultures of trophozoites is shown in **Figures 2** and **3**. Results from zymographies of whole protein extracts of trophozoites (**Figure 2**) revealed protease activity mainly above 70 kDa, with a more intense activity when employing substrate buffer at pH 8.5 (**Figure 2A**). This activity was inhibited by PMSF, indicating the presence of serine proteases.

When substrate buffer of pH 5.0 was employed (**Figure 2B**), *N. fowleri* Limón also showed protease activity between 40 and 70 kDa, not detectable in *N. fowleri* Guanacaste; in this case, a partial inhibition of protease activity by PMSF was observed. The lowest protease activity was evidenced when employing substrate buffer at pH 3.0 (**Figure 2C**), being completely inhibited with PMSF.

Regarding conditioned media, results in **Figure 3** show two clear digestion bands of molecular weights of approximately 140 and 260 kDa when using substrate buffer at pH 8.5. This enzymatic activity was inhibited by PMSF, corresponding to high molecular weight serine proteases. Moreover, the presence of cysteine or metalloproteases was not detected in conditioned media from each of the isolates. For zymographies employing substrate buffers at pH 3.0 and 5.0, no digestion bands were observed in the gels.

### 3.3 Proteomic analyses of excretion/secretion products

**Figure 4:**
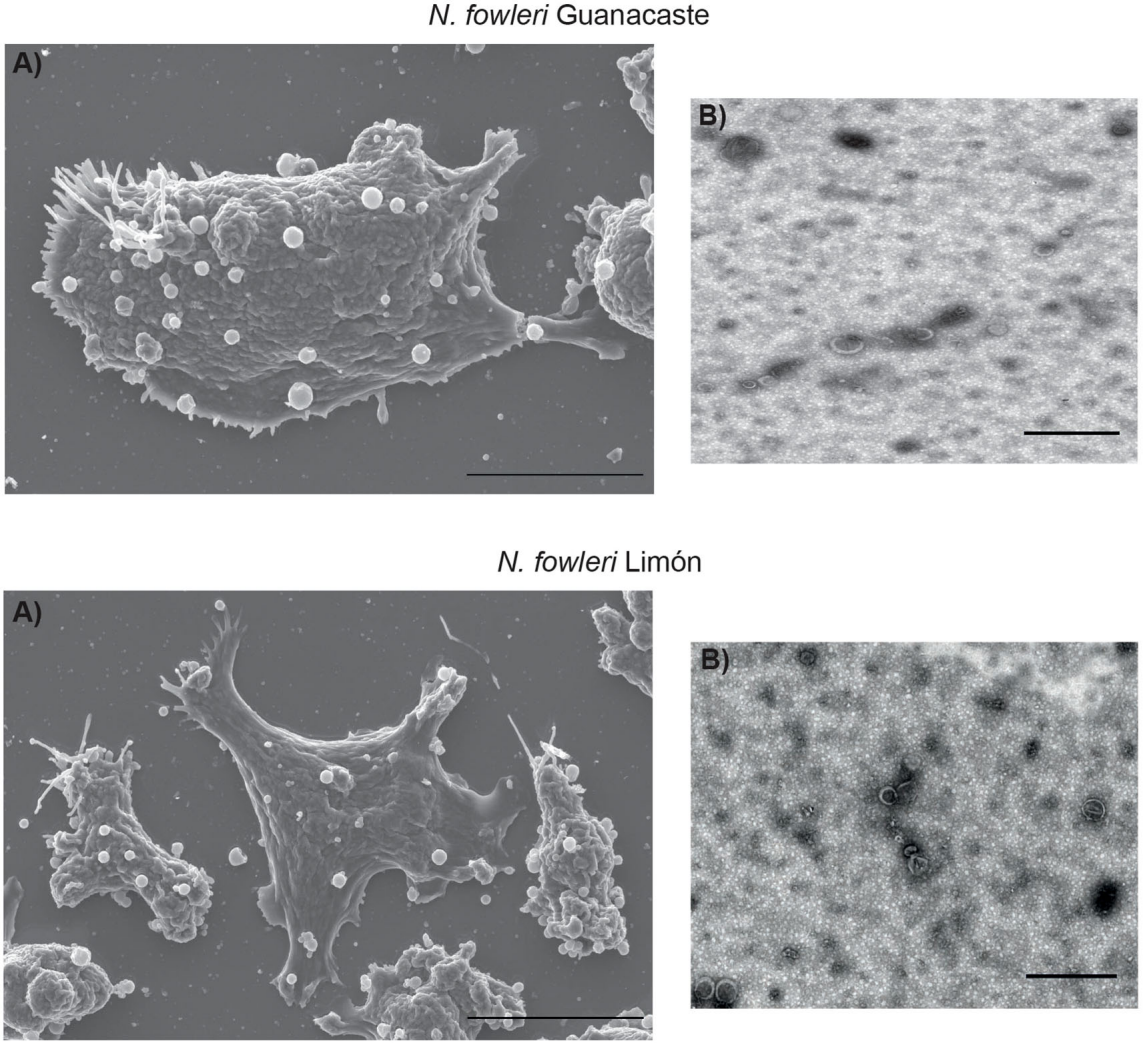
Secretion of extracellular vesicles by trophozoites of *Naegleria fowleri* Guanacaste and *N. fowleri* Limón: **(A)** scanning electron microscopy (scale bar: 10 µm) and **(B)** transmission electron microscopy (scale bar:1 µm).

Conditioned media and EVs of each *N. fowleri* isolate were collected after a five-hour incubation of trophozoites in 2% casein hydrolysate culture medium without serum nor antibiotics. EVs were isolated by ultracentrifugation, and characterized as previously described (Retana Moreira et al., 2022; 2024). **Figure 4** shows EVs secreted by *N. fowleri* trophozoites.

**Figure 5:**
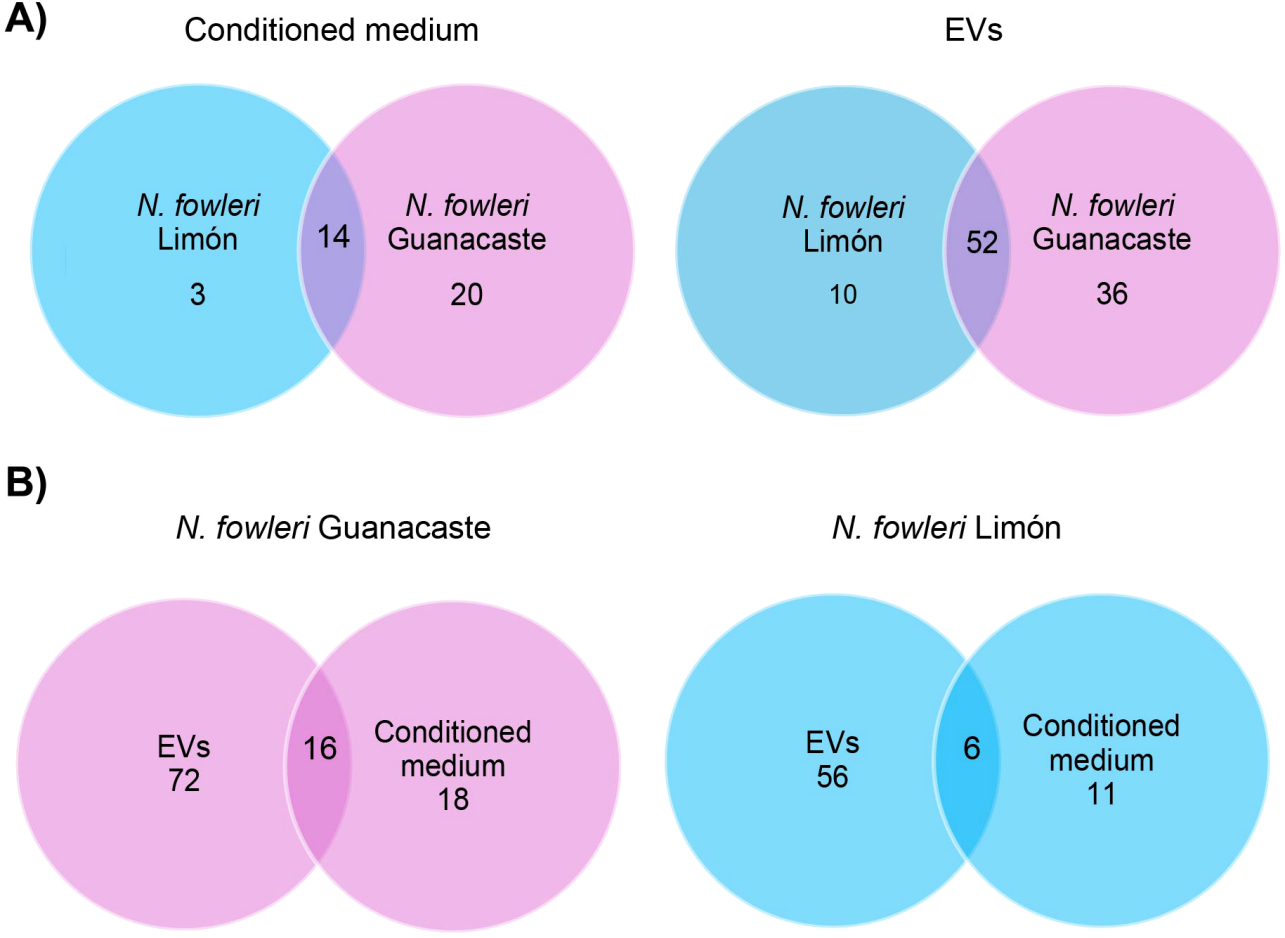
Venn diagrams of specific and shared proteins between conditioned media and EVs of each *Naegleria fowleri* isolate: **(A)** comparison between conditioned media and EVs of the isolates, and **(B)** comparison between conditioned media and EVs for each isolate.

Proteins identified in conditioned media and EVs from each *N. fowleri* isolate are summarized in **Figure 5** and **Supplementary Tables 1-4**. A total of 88 and 62 non-redundant proteins (≥ 1 matched unique peptides) were found as part of the cargo of EVs secreted by trophozoites of *N. fowleri* Guanacaste and *N. fowleri* Limón, while conditioned media results revealed 34 and 17 non-redundant proteins, respectively. It is important to highlight the high number of uncharacterized proteins, especially for the case of EVs (91 proteins for Guanacaste isolate, and 72 proteins for Limón isolate).

Most abundant proteins identified in conditioned media and shared by both *N. fowleri* isolates are shown in **Table 1**. In EVs, peptidases, lipases, cathepsin B, fowlerpain and actin were included within the top of most abundant proteins.

**Table 1:** Most abundant proteins in conditioned media, identified by mass spectrometry, in both isolates of *Naegleria fowleri*.

Protein group	Accession	Avg. Mass	Description
7	A0A6A5BE73	299459	HP domain-containing protein
3	B5M6J9	41726	Actin (Fragment)
8	A0A6A5CAR5	48776	Elongation factor 1-alpha
21	A0A6A5C551	66270	Calponin-homology (CH) domain-containing protein
27	A0A6A5BU22	33586	DUF1394 domain-containing protein
23	Q6B3P1	71408	Hsp70
22	A0A6A5BVM1	113925	Ras-GAP domain-containing protein
18	A0A6A5CCE5	69452	SHOCT domain-containing protein
12	A0A6A5BKQ4	105232	EGF-like domain-containing protein
19	A0A6A5BF81	38294	Fructose-bisphosphate aldolase

**Figure 6:**
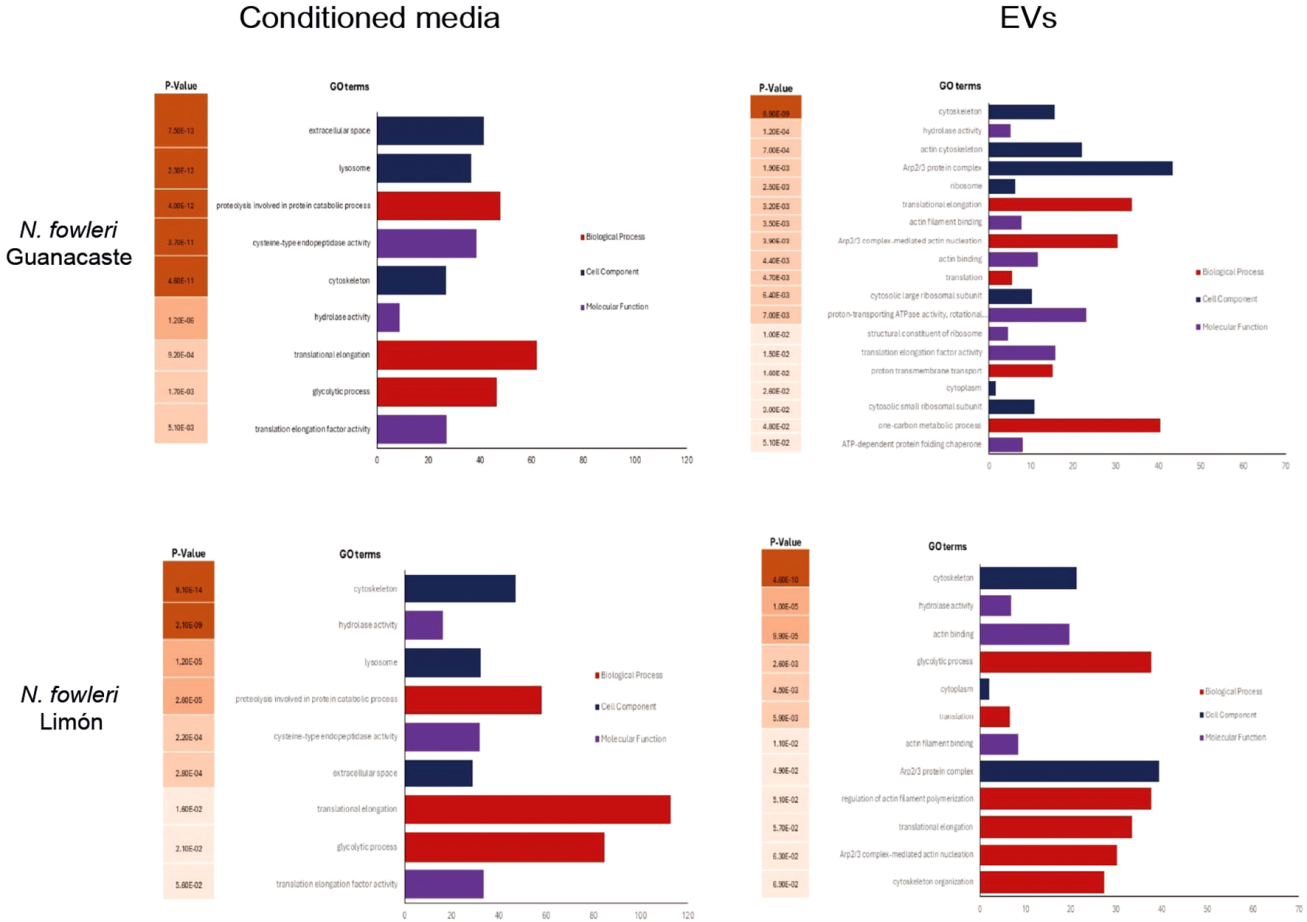
Gene ontology terms enrichment analysis of proteins of conditioned media and EVs of *Naegleria fowleri* Guanacaste and *N. fowleri* Limón, categorized by molecular function (*p* ≤ 0.01).

Following a gene ontology (GO) analysis, the most represented GO term in the category biological processes in the *N. fowleri* Guanacaste proteins were assigned as “translational elongation” and “Arp2/3 complex-mediated actin nucleation”. Similarly, the most represented GO terms within the category molecular function were “hydrolase activity” and “actin filament binding”. For the category “cell component”, the most represented GO terms were “cytoskeleton”, “actin cytoskeleton” and “Arp2/3 protein complex” (**Figure 6**). Regarding *N. fowleri* Limón, the most represented GO terms in the category biological processes were “glycolytic process” and “translation”, while for the category “molecular function”, the most represented GO terms were “hydrolase activity” and “actin filament binding”. Finally, for “cell component”, the most represented GO terms were “cytoskeleton” and “cytoplasm” (**Figure 6**).

Results of conditioned media revealed “proteolysis involved in protein catabolic process” and “hydrolase activity” as the most represented GO terms in the categories biological and molecular function, respectively, for both isolates. Within the category cell component, “extracellular space” and “lysosome” were the most represented GO terms for *N. fowleri* Guanacaste, while “cytoskeleton” and “lysosome” were the most represented GO terms for *N. fowleri* Limón (**Figure 6**).

### 3.4 Evaluation of the cytopathic effect of trophozoites and conditioned media

**Figure 7:**
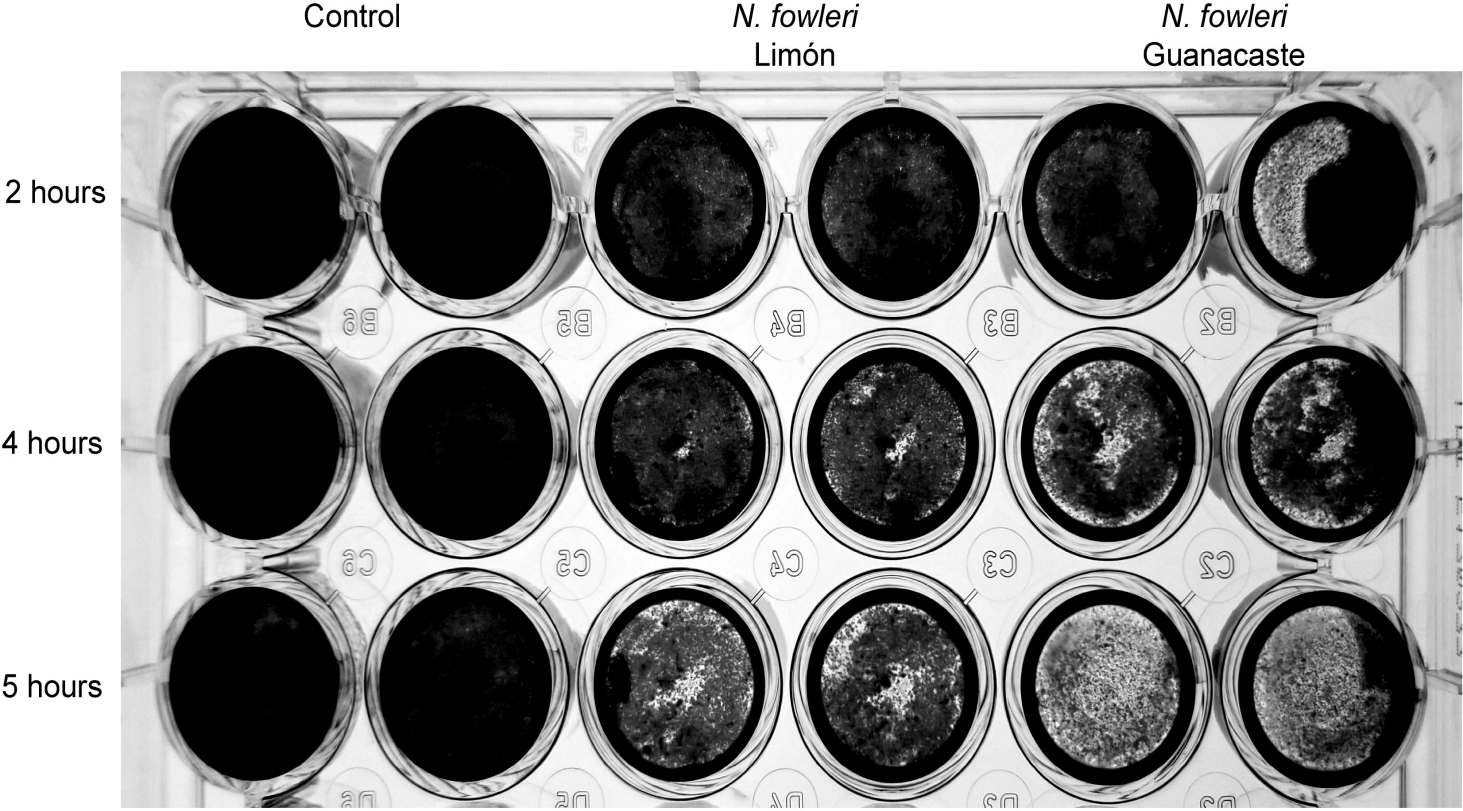
Crystal violet staining that shows destruction of Vero cell monolayers by trophozoites of *Naegleria fowleri* Guanacaste and *N*. *fowleri* Limón. For these experiments, 2.5 x 10^5^ cells/well were seeded until confluence and 1.25 x 10^5^ trophozoites/well were added and incubated for five hours at 37 °C. The destruction of the cell monolayer was recorded for two, four and five hours.

**Figure 7** shows results from cytopathic effect after crystal violet staining. From this Figure, it was demonstrated that the destruction of the cell monolayers started at two hours post infection. Moreover, a more rapid effect was observed when incubating the cells with trophozoites of *N. fowleri* Guanacaste.

Cytopathic effect was also analyzed by LDH release assay. In this case, results obtained after the incubation with trophozoites indicated that damage started after three hours of incubation, with a sustained increase in lactate dehydrogenase released until the end of the experiment (five hours) (**Figure 7**). In these experiments, *N. fowleri* Guanacaste presented a significantly higher percentage of cytotoxicity (23.0%) when compared to *N. fowleri* Limón (10.3%), percentages obtained at the last hour of incubation. On the other hand, when conditioned media were employed, increases in LDH release into the medium were not observed over time.

Experiments performed to evaluate the effect of conditioned media and EVs of each *N. fowleri* isolate over Vero cell viability revealed no significant effects in cells after the incubation for 24 hours, since the reduction of Presto Blue™ reagent was observed in all the wells.

### 3.5 Drug susceptibility assays

**Table 2** shows cytotoxic concentration 50% of miltefosine and amphotericin B against *N. fowleri* Guanacaste and *N. fowleri* Limón isolates. In these experiments, no statistically significant differences were observed between CC50 of both isolates with either drug (*p* > 0.05). In the case of voriconazole, 50% inhibition was not achieved by any of the isolates.

**Table 2:** Cytotoxic concentration 50 (CC50) of amphotericin B and miltefosine over trophozoites of *Naegleria fowleri* Guanacaste and *N*. *fowleri* Limón.

Isolate	Drug	
	Amphotericin B	Miltefosine
*Naegleria fowleri* Guanacaste	15,62 µg/mL	2,5 µg/mL
*Naegleria fowleri* Limón	31,25 µg/mL	2,5 µg/mL

## 4 Discussion

In this work, a characterization and comparative analysis between the first two clinical isolates of *N. fowleri* (Guanacaste and Limón), recovered from two cases of PAM diagnosed in Costa Rica in 2020, is presented. Analyses included protein and proteases profiles, proteomic analyses of excretion/secretion products, evaluation of the cytopathic effect and drug susceptibility assays. In general, the results obtained were similar for both isolates, especially in protein profiles and cargoes. However, slight differences in protease profiles and cytopathic effect were also found.

Protein profiles of trophozoites resulted remarkably similar between both isolates. In this sense, the presence of multiple protein bands between 10 and 250 KDa was observed; we highlight the presence of multiple bands below 60 kDa (**Figure 1**). Protein patterns obtained in these analyses were also similar to others previously reported (Pernin et al., 1983; Serrano Luna et al., 2007; Flores-Huerta et al., 2019; Retana Moreira et al., 2022).

In the case of whole protein extracts of trophozoites, protease activity was evidenced at every pH tested, being more intense at pH 8.5, but very limited at pH 3.0. Serine proteases were the main responsible for this activity, since the highest inhibition was observed after the incubation with PMSF (**Figure 2**). The effect of pH on proteolytic patterns suggests that amoebae could have a variety of protease activities differing in pH optimum, ranging from acid to alkaline, and this variation may be important for the amoeba to gain access to various nutrients in different environments, including human tissues (Serrano Luna et al., 2007). We highlight protease activity under 100 KDa, identified at pH 5.0, only in the case of *N. fowleri* Limón. This activity could correspond to acidic proteases, since its optimal activity is evidenced at pHs 2.0 - 5.0. Proteases of this type include aspartic proteases like pepsin, renin, and cathepsin D, the same that have been described in other *Naegleria* isolates (Vyas, 2014). Since the pattern of peptidase activity is not useful for discriminating between highly pathogenic and low pathogenic *N. fowleri* (Vyas, 2014), more studies are necessary to determine if this difference could have a role in other biological aspects of this type of microorganisms.

In contrast to the previously mentioned results, protease activity of conditioned media was evident only when employing the substrate buffer at pH 8.5. The presence of a higher protease activity in whole protein extracts of trophozoites could be related to the possible presence of these enzymes in a compartmentalized way within *N. fowleri*, including in the membrane fraction (as free proteins or/and in extracellular vesicles), so that its release could be facilitated upon contact of the amoeba with a substrate, as well as during the infection process (Vyas, 2014).

Previous studies performed by our research group also revealed protease activity, mainly consisting of serine proteases, in EVs secreted by trophozoites of *N. fowleri*; a minimal presence of cysteine proteases was also found (Retana Moreira et al., 2022). This result is similar to what has been described for clinical and environmental isolates of *Acanthamoeba* (Gonçalves et al., 2018, 2019; Lin et al., 2019; Costa et al., 2021). However, these results also differ from other reports regarding *N. fowleri*, in which a predominance of cysteine proteases has been detected (Serrano Luna et al., 2007; Vyas et al., 2015), although serine proteases were also identified. In free living amoebae, protease activity is considered a virulence factor (Serrano Luna et al., 2007), as it is involved in tissue destruction and in pathogenesis. In this sense, the secretion and release of these enzymes have been related to increases in mitochondrial and cell permeability, extracellular matrix degradation, induction of apoptosis and cell death (Visvesvara et al., 2007; Betanzos et al., 2019). For example, in the specific case of serine proteases, it has been suggested an important role in extracellular matrix degradation during the initial stages of an infection with *Acanthamoeba*, facilitating tissue invasion (Khan et al., 2000; Alsam et al., 2005; Omaña Molina et al., 2013).

Proteomic analyses performed in this study revealed a higher number of proteins in conditioned media of *N. fowleri* Guanacaste than in conditioned media of *N. fowleri* Limón (64 vs. 21 proteins); the same trend was observed in EVs samples (83 vs. 59 proteins, respectively). Despite this difference, more than 80% of the identified proteins in both types of samples were shared between both isolates (**Figure 5**). Additionally, it was also evident the difference in numbers of proteins between EVs and conditioned media for the same isolate. From these results, we could hypothesize that this difference could have a methodological explanation, since a concentration process occurs during the ultracentrifugation steps for EVs recovery. Despite this difference in protein numbers between EVs and conditioned media, more than 45% of the identified proteins in conditioned media were part of the EVs cargo (**Figure 5**).

The presence of actin, cathepsins, dehydrogenases, aldolases, lipases, ceramidases, elongation factor 1-alpha and fowlerpain, among others, in EVs and conditioned media, suggests a possible role in pathogenesis during an infection with *N. fowleri* (especially for EVs). As examples, it is already known that actin is an essential protein for all eukaryotic cells, in which the actin cytoskeleton forms dynamic filaments (Gutiérrez-Sánchez et al., 2020); it also plays fundamental structural roles and participates in many cellular processes, including phagocytosis and cell motility. In *N. fowleri*, actin is in the cytoplasm, pseudopods and particularly in food-cups, structures that play a key role during the invasion, migration, and phagocytosis processes (Gutiérrez-Sánchez et al., 2020). HSP-70 is another protein constantly found in EVs of *N. fowleri* (Retana Moreira et al., 2022; Russell et al., 2023; Rodríguez Mera et al., 2024), and its presence also on the surface of trophozoites may be related to adhesion, contributing to *N. fowleri* invasion and migration to the brain, as suggested by Flores-Suárez et al. (2024). Elongation factor 1-alpha (eeEF1 α) is a protein that has also been found in exosomes secreted by *Leishmania* in early infections and identified as a crucial factor for immunosuppression and priming of host cells for *Leishmania* invasion (Silverman and Reiner, 2012; Timm et al., 2017), a fact that could suggest it may have a similar role in infections with *N. fowleri*.

**Figure 8:**
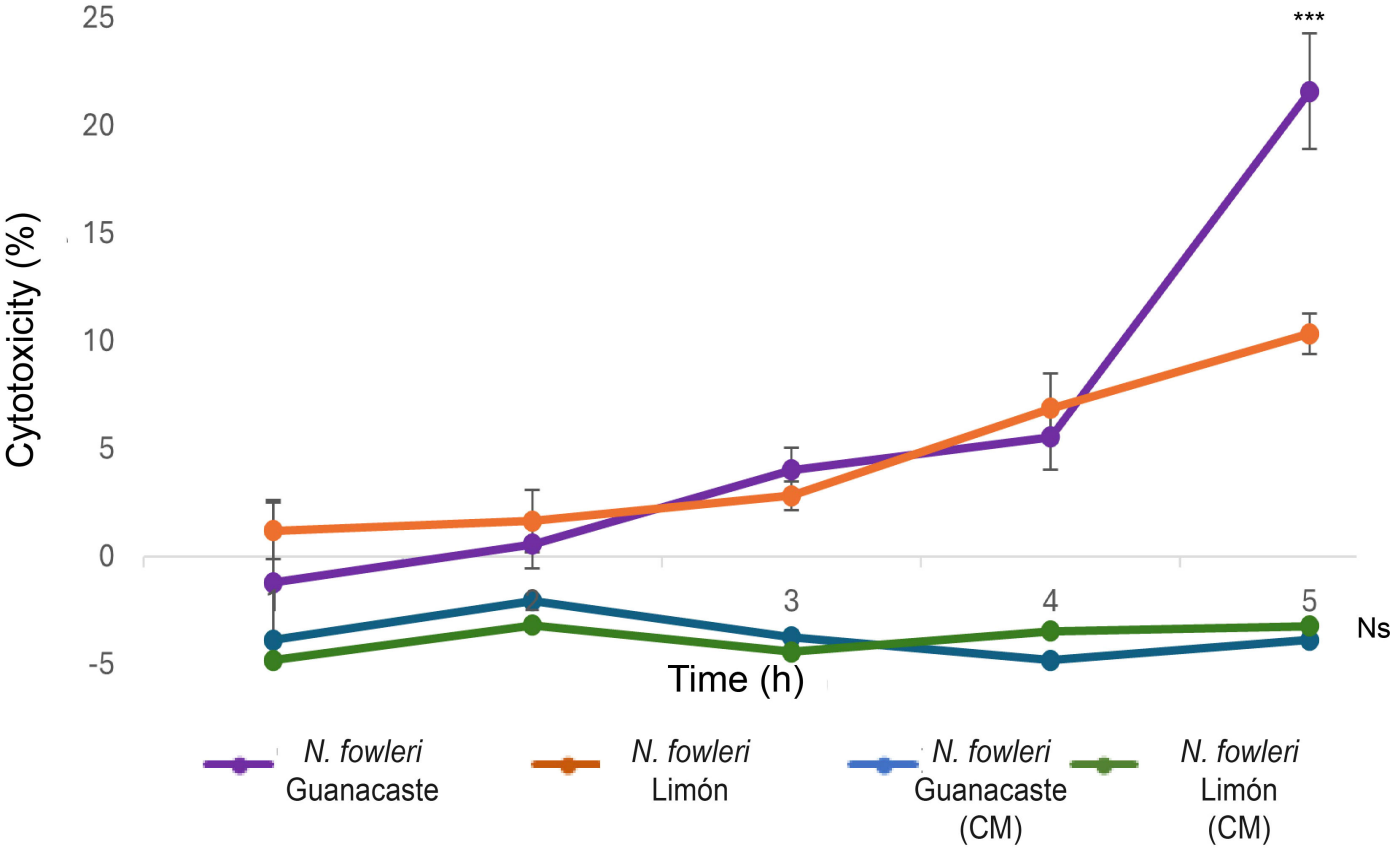
Lactate dehydrogenase release assay to evidence of cell death after the incubation of trophozoites and conditioned media (CM) of *Naegleria fowleri* Guanacaste and *N*. *fowleri* Limón with Vero cells for five hours. Cell death was quantified by LDH release into the medium due to cell lysis. Significant differences between trophozoites of both isolates were found only at five hours of incubation (***, *p* < 0.05). Moreover, no significant differences were observed for conditioned media of both isolates, nor differences in incubation times (Ns, *p* > 0.05).

Comparisons performed to evaluate differences in cytopathic effect of the two *N. fowleri* isolates are shown in **Figure 7**. From the Figure, it was possible to observe damage to the monolayer after two hours of incubation with trophozoites of *N. fowleri* Guanacaste isolate. These results were consistent with results obtained from the lactate dehydrogenase assay, which revealed LDH release after three hours of incubation, with a sustained increase until the end of the experiment (**Figure 8**) that was higher when incubating the cells with *N. fowleri* Guanacaste. Other research groups have conducted similar studies but using other species of pathogenic free-living amoebae like *Acanthamoeba* sp. and *Balamuthia mandrillaris*. For the case of *Acanthamoeba* sp., the authors demonstrated that some of the genotypes exert cell destruction over much longer periods, ranging from 24 to 240 hours (González Robles et al., 2014). For *B. mandrillaris*, it has been reported that cell damage begins from four to eight days, reaching destruction up to 16 days later through a pyknosis mechanism (Yera et al., 2015). Our results demonstrate the high virulence of *N. fowleri* and suggest that amoebae cause contact-dependent cell destruction, since there was an increase in LDH release over time when Vero cells were incubated with trophozoites, but not with conditioned media (at least when employing the concentration assayed in this study). Moreover, conditioned media and EVs do not seem to directly affect cell viability since Vero cells were metabolically active and reduced PrestoBlue™, a result that coincides with a previous report in literature, in which viability of different cell lines was not affected after coming into contact with different doses of EVs (0.675 µg – 10 µg) secreted by trophozoites of *N. fowleri* (Russell et al., 2023), demonstrated using a real time kit. In this work, although both isolates showed a similar “cell-killing” capacity *in vitro*, Guanacaste isolate seems to be more aggressive during the first five hours post infection. Experiments using an *in vivo* model are necessary to determine whether this trend is reflected in the time and mortality rate of animals.

Results from drug susceptibility tests showed that both miltefosine and amphotericin B have therapeutic activity against the autochthonous isolates of *N. fowleri* (**Table 1**), with no evidence of differences in susceptibility for these drugs. For miltefosine, both isolates were susceptible to the last dilution evaluated (2.5 µg/mL), being lower than the ones reported in the literature and suggesting that this drug is highly effective and could be considered in the therapeutic scheme for PAM cases in Costa Rica. For amphotericin B, the cytotoxic concentration 50 obtained was 15.62 µg/mL for Guanacaste isolate and 31.25 µg/mL for Limón isolate, much higher than the average reported in literature. Voriconazole did not reach an *in vitro* inhibitory concentration 50% against any of the isolates. In this sense, higher concentrations should be assessed; however, difficulties due to the low solubility of these compounds in water do not allow the evaluation of these concentrations.

*In vitro* studies have determined minimal inhibitory concentrations (MICs) of 0.78 µg/mL for amphotericin B when using reference strains (Kim et al., 2008), as well as values ​​between 25 µg/mL and 40 µM (36.96 µg/mL) for miltefosine (Schuster et al., 2006; Kim et al., 2008). Recently, Russell and Kyle (2022) determined MICs of different drugs for clinical isolates of *N. fowleri* and reported highly variable values ​​between 0.63 µM (0.15 µg/mL) and 1.73 µM (1.60 µg/mL) for amphotericin B, as well as between 18.6 µM (7.6 µg/mL) and 116 µM (47 µg/mL) for miltefosine. Results obtained for amphotericin B represent a worrying scenario since this drug is the only one approved by the Centers for Disease Control and Prevention as a first-line treatment for PAM and has been used in all survival cases, although the survival rate has been only 5%. Moreover, the use of this drug is controversial because it does not target anything specific to the amoeba but rather participates in the lysis of cell membranes by interacting with their sterols (both in fungi and parasites). Additionally, this drug has low solubility, which affects its absorption, bioavailability and elimination (Martín Escolano et al., 2021).

It is important to mention that most drug susceptibility research has been conducted with older isolates or with ATCC reference strains of *N. fowleri*, since there are few clinical isolates of this microorganism in the world, including those employed in this work. Reference strains maintained under controlled laboratory conditions may show discrepant behavior and decreased virulence, compared to wild strains found in natural sources. Therefore, obtaining results that better reflect the reality of the pathogenicity of this amoeba in nature is valuable to better direct the prospection of new therapeutic strategies.

Characterization of the different isolates of *Naegleria fowleri*, including its excretion/secretion products, is crucial for a better understanding of the virulence and pathogenicity of these amoeba, and could help to explain whether different isolates differ in the severity or course of primary acute meningoencephalitis. Moreover, results from this type of investigations could definitely provide targets for the development of novel diagnostic and therapeutic options, critical for the effective management of this life-threatening infection.

## Data Availability

Mass spectrometry data were deposited to the ProteomeXchange Consortium via the MassIVE repository with the dataset identifier PXD063517.
