# Supplementary material for: Characterization of Naegleria fowleri from two human cases: insights into its excretion/secretion products
Source: Front Cell Infect Microbiol. 2025 May 6;15:1585448. doi: 10.3389/fcimb.2025.1585448 (PMC12089049; doi:10.3389/fcimb.2025.1585448)
Supplement: Supplementary file 1 [file Table1.docx]

Supplementary Material

# Supplementary Table 1. Non redundant proteins in extracellular vesicles secreted by trophozoites of *Naegleria fowleri* Guanacaste

| **Protein Group** | **Accession #** | **#Unique peptide** | **Avg. Mass** | **Description** |
| --- | --- | --- | --- | --- |
| 5 | A0A6A5CAR5 | 18 | 48776 | Elongation factor 1-alpha |
| 2 | B5M6J9 | 15 | 41726 | Actin (Fragment) |
| 18 | A0A6A5C551 | 12 | 66270 | Calponin-homology (CH) domain-containing protein |
| 21 | Q6B3P1 | 12 | 71408 | Hsp70 |
| 27 | A0A6A5BVM1 | 11 | 113925 | Ras-GAP domain-containing protein |
| 30 | A0A6A5CCE5 | 10 | 69452 | SHOCT domain-containing protein |
| 13 | A0A4V8H039 | 9 | 36595 | Glyceraldehyde-3-phosphate dehydrogenase |
| 34 | A0A6A5BE73 | 9 | 299459 | HP domain-containing protein |
| 36 | A0A6A5BPW6 | 9 | 68559 | Rho-GAP domain-containing protein |
| 48 | A0A6A5BU22 | 8 | 33586 | DUF1394 domain-containing protein |
| 31 | A0A6A5BKQ4 | 8 | 105232 | EGF-like domain-containing protein |
| 17 | A0A6A5BF81 | 8 | 38294 | Fructose-bisphosphate aldolase |
| 35 | A0A6A5BYK8 | 7 | 51810 | Adenosylhomocysteinase |
| 35 | A0A1Z0YU84 | 7 | 51752 | Adenosylhomocysteinase |
| 25 | A0A6A5CIW0 | 7 | 50682 | Non-specific serine/threonine protein kinase |
| 53 | A0A6A5BN25 | 6 | 33208 | 14_3_3 domain-containing protein |
| 32 | A0A6A5BJJ6 | 6 | 60814 | Coronin |
| 51 | A0A6A5CGD3 | 6 | 38854 | Guanine nucleotide-binding protein subunit beta-like protein |
| 50 | A0A2R2JFX6 | 6 | 50101 | Rab GDP dissociation inhibitor |
| 24 | A0A6A5C539 | 6 | 44084 | WH2 domain-containing protein |
| 57 | A0A6A5CGC6 | 5 | 69772 | Abhydrolase_4 domain-containing protein |
| 58 | A0A6A5BBU7 | 5 | 54111 | Adenylyl cyclase-associated protein |
| 49 | A0A6A5C6Q4 | 5 | 64041 | Lipase_3 domain-containing protein |
| 45 | A0A6A5BPM9 | 5 | 21652 | Thioredoxin domain-containing protein |
| 62 | C6L6E3 | 4 | 34514 | Amino acid decarboxylase (Fragment) |
| 64 | A0A6A5BN74 | 4 | 108689 | Phosphatidylinositol-3,4,5-trisphosphate 3-phosphatase |
| 88 | A0A6A5C150 | 3 | 83351 | C2 domain-containing protein |
| 10 | Q95UJ2 | 3 | 19932 | Membrane protein |
| 66 | A0A6A5BXE3 | 3 | 26208 | Protoglobin domain-containing protein |
| 67 | A0A6A5BXV3 | 3 | 27389 | SCP domain-containing protein |
| 87 | A0A6A5BPK2 | 3 | 176271 | Vitellogenin domain-containing protein |
| 86 | A0A6A5CH81 | 2 | 26142 | 40S ribosomal protein S3a |
| 84 | A0A6A5BMI3 | 2 | 44324 | 4-hydroxyphenylpyruvate dioxygenase |
| 120 | D2V5T7 | 2 | 24163 | 60S ribosomal protein L13 |
| 126 | A0A6A5C126 | 2 | 22000 | 60S ribosomal protein L18a |
| 116 | A0A6A5BJ08 | 2 | 42807 | Actin-related protein 2/3 complex subunit |
| 127 | A0A6A5BLA8 | 2 | 15668 | ADF-H domain-containing protein |
| 113 | A0A6A5BLK1 | 2 | 98186 | AMP-binding domain-containing protein |
| 96 | A0A6A5BZ15 | 2 | 35633 | Arp2/3 complex 34 kDa subunit |
| 78 | A0A6A5BHL2 | 2 | 149735 | CCT-alpha |
| 97 | A0A6A5BXV6 | 2 | 83102 | Cell surface protease |
| 122 | A0A6A5BZL5 | 2 | 80585 | HATPase_c domain-containing protein |
| 131 | A0A6A5BR74 | 2 | 35799 | Malate dehydrogenase |
| 106 | D2W6P3 | 2 | 18962 | Methionine adenosyltransferase (Fragment) |
| 119 | D2W159 | 2 | 118785 | Myosin |
| 98 | D2V5X3 | 2 | 111328 | Penicillin amidase family protein |
| 107 | A0A6A5BNP4 | 2 | 18201 | Peptidyl-prolyl cis-trans isomerase |
| 90 | A0A6A5BH70 | 2 | 101938 | PNPLA domain-containing protein |
| 101 | A0A6A5BUU1 | 2 | 39475 | Protein kinase domain-containing protein |
| 89 | A0A6A5C721 | 2 | 305529 | RGS domain-containing protein |
| 75 | D2VUQ3 | 2 | 20623 | Rho family small GTPase |
| 106 | A0A6A5BQ69 | 2 | 42079 | S-adenosylmethionine synthase |
| 104 | A0A6A5BV24 | 2 | 93499 | Tr-type G domain-containing protein |
| 109 | A0A6A5BRS8 | 2 | 37733 | t-SNARE coiled-coil homology domain-containing protein |
| 117 | A0A6A5BSQ0 | 2 | 75432 | Ubiquitin-like domain-containing protein |
| 162 | D2V160 | 1 | 16265 | 40S ribosomal protein S16 |
| 190 | A0A6A5BIS7 | 1 | 28689 | 40S ribosomal protein SA |
| 124 | D2VU93 | 1 | 17642 | 60S ribosomal protein L40 |
| 180 | A0A6A5CBU0 | 1 | 49751 | 60S ribosomal protein L6 |
| 177 | A0A6A5C6A3 | 1 | 29343 | 60S ribosomal protein L7a |
| 175 | A0A6A5BJ87 | 1 | 58011 | AAA domain-containing protein |
| 182 | A0A6A5BNJ8 | 1 | 19774 | Actin-related protein 2/3 complex subunit 4 |
| 179 | A0A6A5BR52 | 1 | 113716 | AP-2 complex subunit alpha |
| 137 | A0A6A5BGR0 | 1 | 79840 | ARID domain-containing protein |
| 111 | A0A6A5C7K8 | 1 | 59142 | CYTOSOL_AP domain-containing protein |
| 155 | A0A6A5BIP7 | 1 | 50398 | DUF4476 domain-containing protein |
| 82 | D2VJ04 | 1 | 491424 | Dynein-1-alpha heavy chain, flagellar inner arm I1 complex |
| 154 | A0A6A5CFS9 | 1 | 119818 | Ephrin_rec_like domain-containing protein |
| 176 | A0A6A5CB00 | 1 | 79044 | Formyltetrahydrofolate synthetase |
| 144 | A0A1L1XWF9 | 1 | 33944 | Fowlerpain-2 |
| 178 | A0A6A5BSI5 | 1 | 24624 | GTP-binding nuclear protein |
| 125 | A0A6A5BFJ4 | 1 | 67844 | H(+)-transporting two-sector ATPase |
| 193 | A0A6A5B8R0 | 1 | 143225 | HECT domain-containing protein |
| 168 | A0A6A5BQM0 | 1 | 53450 | Isocitrate dehydrogenase [NADP] |
| 157 | A0A6A5BD85 | 1 | 41008 | Mitogen-activated protein kinase |
| 192 | A0A6A5BWT5 | 1 | 36791 | Peptidase S1 domain-containing protein |
| 194 | D2W4M3 | 1 | 58924 | Predicted protein |
| 76 | D2V1N7 | 1 | 26562 | Rab family small GTPase |
| 80 | D2UX92 | 1 | 23389 | Ras family small GTPase (RAP-1) |
| 69 | A0A1Z0YU85 | 1 | 22430 | Ras-related c3 botulinum toxin substrate 1 isoform x2 |
| 149 | A0A6A5C514 | 1 | 41361 | Ribos_L4_asso_C domain-containing protein |
| 147 | A0A6A5CGQ8 | 1 | 32008 | S5 DRBM domain-containing protein |
| 166 | A0A6A5BZP8 | 1 | 36267 | Smr domain-containing protein |
| 83 | A0A6A5BYP8 | 1 | 124677 | TLDc domain-containing protein |
| 124 | Q25558 | 1 | 13426 | Ubiquitin (Fragment) |
| 145 | D2V5W7 | 1 | 52721 | Vacuolar proton pump subunit B |
| 41 | A0A6A5C6W5 | 1 | 17616 | V-type proton ATPase proteolipid subunit |
| 186 | A0A6A5BLL9 | 1 | 92920 | V-type proton ATPase subunit |

# Supplementary Table 2. Non redundant proteins in extracellular vesicles secreted by trophozoites of

# *Naegleria fowleri* Limón

| **Protein Group** | **Accession** | **#Unique peptide** | **Avg. Mass** | **Description** |
| --- | --- | --- | --- | --- |
| 7 | A0A6A5BE73 | 23 | 299459 | HP domain-containing protein |
| 3 | B5M6J9 | 13 | 41726 | Actin (Fragment) |
| 8 | A0A6A5CAR5 | 13 | 48776 | Elongation factor 1-alpha |
| 21 | A0A6A5C551 | 12 | 66270 | Calponin-homology (CH) domain-containing protein |
| 27 | A0A6A5BU22 | 11 | 33586 | DUF1394 domain-containing protein |
| 23 | Q6B3P1 | 10 | 71408 | Hsp70 OS=Naegleria fowleri |
| 22 | A0A6A5BVM1 | 10 | 113925 | Ras-GAP domain-containing protein |
| 18 | A0A6A5CCE5 | 10 | 69452 | SHOCT domain-containing protein |
| 12 | A0A6A5BKQ4 | 9 | 105232 | EGF-like domain-containing protein |
| 19 | A0A6A5BF81 | 9 | 38294 | Fructose-bisphosphate aldolase |
| 36 | A0A6A5BPW6 | 9 | 68559 | Rho-GAP domain-containing protein |
| 10 | A0A4V8H039 | 8 | 36595 | Glyceraldehyde-3-phosphate dehydrogenase |
| 26 | A0A6A5CIW0 | 8 | 50682 | Non-specific serine/threonine protein kinase |
| 37 | A0A6A5BN25 | 7 | 33208 | 14_3_3 domain-containing protein |
| 38 | A0A6A5BN74 | 7 | 108689 | Phosphatidylinositol-3,4,5-trisphosphate 3-phosphatase |
| 29 | A0A6A5C539 | 7 | 44084 | WH2 domain-containing protein |
| 33 | A0A6A5BJJ6 | 6 | 60814 | Coronin |
| 44 | A0A6A5CGD3 | 6 | 38854 | Guanine nucleotide-binding protein subunit beta-like protein |
| 45 | A0A6A5C721 | 6 | 305529 | RGS domain-containing protein |
| 39 | A0A6A5CGC6 | 5 | 69772 | Abhydrolase_4 domain-containing protein |
| 48 | A0A6A5BYK8 | 5 | 51810 | Adenosylhomocysteinase |
| 20 | Q95UJ2 | 4 | 19932 | Membrane protein |
| 57 | A0A2R2JFX6 | 3 | 50101 | Rab GDP dissociation inhibitor |
| 90 | A0A6A5BMI3 | 2 | 44324 | 4-hydroxyphenylpyruvate dioxygenase |
| 100 | A0A6A5C126 | 2 | 22000 | 60S ribosomal protein L18a |
| 92 | A0A6A5BJ08 | 2 | 42807 | Actin-related protein 2/3 complex subunit |
| 95 | A0A6A5BBU7 | 2 | 54111 | Adenylyl cyclase-associated protein |
| 94 | A0A6A5C150 | 2 | 83351 | C2 domain-containing protein |
| 83 | A0A6A5BXV6 | 2 | 83102 | Cell surface protease |
| 84 | A0A6A5CB00 | 2 | 79044 | Formyltetrahydrofolate synthetase |
| 80 | A0A6A5BR74 | 2 | 35799 | Malate dehydrogenase |
| 64 | D2V5X3 | 2 | 111328 | Penicillin amidase family protein |
| 76 | D2VUQ3 | 2 | 20623 | Rho family small GTPase |
| 86 | A0A6A5BRS8 | 2 | 37733 | t-SNARE coiled-coil homology domain-containing protein |
| 110 | A0A6A5CH81 | 1 | 26142 | 40S ribosomal protein |
| 140 | D2V5T7 | 1 | 24163 | 60S ribosomal protein L13 |
| 139 | A0A6A5BV84 | 1 | 93169 | ABC transporter domain-containing protein |
| 130 | A0A6A5BNF3 | 1 | 15490 | ADF-H domain-containing protein |
| 137 | C6L6E3 | 1 | 34514 | Amino acid decarboxylase (Fragment) |
| 148 | A0A6A5CBJ9 | 1 | 85170 | AMP-binding domain-containing protein |
| 115 | A0A6A5BZ15 | 1 | 35633 | Arp2/3 complex 34 kDa subunit |
| 111 | A0A6A5C1N3 | 1 | 34615 | Calponin-homology (CH) domain-containing protein |
| 66 | A0A6A5BHL2 | 1 | 149735 | CCT-alpha |
| 87 | A0A6A5C7K8 | 1 | 59142 | CYTOSOL_AP domain-containing protein |
| 113 | A0A6A5BPN7 | 1 | 122681 | DH domain-containing protein |
| 129 | D2VGT4 | 1 | 34856 | Methenyltetrahydrofolate cyclohydrolase |
| 102 | D2W159 | 1 | 118785 | Myosin |
| 104 | A0A6A5BH70 | 1 | 101938 | PNPLA domain-containing protein |
| 145 | D2W4M3 | 1 | 58924 | Predicted protein |
| 125 | A0A6A5C9M8 | 1 | 68972 | Pre-mRNA-splicing factor SLU7 |
| 93 | A0A6A5BUU1 | 1 | 39475 | Protein kinase domain-containing protein |
| 68 | D2V1N7 | 1 | 26562 | Rab family small GTPase |
| 69 | D2UX92 | 1 | 23389 | Ras family small GTPase (RAP-1) |
| 59 | A0A1Z0YU85 | 1 | 22430 | Ras-related c3 botulinum toxin substrate 1 isoform x2 |
| 118 | A0A6A5C514 | 1 | 41361 | Ribos_L4_asso_C domain-containing protein |
| 70 | A0A6A5BXV3 | 1 | 27389 | SCP domain-containing protein |
| 126 | A0A6A5BZP8 | 1 | 36267 | Smr domain-containing protein |
| 127 | A0A6A5BWU3 | 1 | 27726 | Triosephosphate isomerase |
| 128 | Q25558 | 1 | 13426 | Ubiquitin (Fragment) |
| 124 | A0A6A5BSQ0 | 1 | 75432 | Ubiquitin-like domain-containing protein |
| 98 | D2V5W7 | 1 | 52721 | Vacuolar proton pump subunit B |
| 79 | A0A6A5BPK2 | 1 | 176271 | Vitellogenin domain-containing protein |
| 47 | A0A6A5C6W5 | 1 | 17616 | V-type proton ATPase proteolipid subunit |

# Supplementary Table 3. Non redundant proteins in conditioned medium of *Naegleria fowleri*

# Guanacaste

| **Protein Group** | **Accession** | **#Unique peptide** | **Avg. Mass** | **Description** |
| --- | --- | --- | --- | --- |
| 6 | A0A6A5C6Q4 | 11 | 64041 | Lipase_3 domain-containing protein |
| 3 | A0A6A5BF81 | 10 | 38294 | Fructose-bisphosphate aldolase |
| 5 | A0A6A5C7K8 | 9 | 59142 | CYTOSOL_AP domain-containing protein |
| 14 | A0A6A5BUH9 | 7 | 53729 | Dihydrolipoyl dehydrogenase |
| 4 | A0A4V8H039 | 7 | 36595 | Glyceraldehyde-3-phosphate dehydrogenase |
| 2 | B5M6J9 | 6 | 41726 | Actin (Fragment) |
| 10 | A0A6A5CEE5 | 5 | 35038 | Pept_C1 domain-containing protein |
| 13 | A0A6A5CD16 | 5 | 245382 | VWFA domain-containing protein |
| 7 | X5D911 | 4 | 34137 | Cathepsin B-like protein |
| 11 | A0A6A5CFU9 | 4 | 42776 | Peptidase A1 domain-containing protein |
| 25 | A0A6A5BYK8 | 3 | 51810 | Adenosylhomocysteinase |
| 26 | A0A6A5CAR5 | 3 | 48776 | Elongation factor 1-alpha |
| 21 | A0A6A5C8E9 | 3 | 65714 | Guanine nucleotide-binding protein subunit beta-like protein |
| 23 | A0A6A5BE22 | 3 | 58093 | Methylmalonate-semialdehyde dehydrogenase (CoA acylating) |
| 15 | A0A6A5BPM9 | 3 | 21652 | Thioredoxin domain-containing protein |
| 26 | A0A6A5CB43 | 3 | 79605 | Tr-type G domain-containing protein |
| 42 | A0A6A5BMI3 | 2 | 44324 | 4-hydroxyphenylpyruvate dioxygenase |
| 36 | A0A6A5BZ92 | 2 | 59536 | BPI2 domain-containing protein |
| 17 | X5D761 | 2 | 38387 | Cathepsin B protein |
| 31 | A0A6A5BJJ6 | 2 | 60814 | Coronin |
| 29 | Q25547 | 2 | 38792 | Cysteine proteinase homolog (Fragment) |
| 1 | A0A1L1XWF9 | 2 | 33944 | Fowlerpain-2 |
| 24 | A0A6A5BD42 | 2 | 82209 | Neutral ceramidase |
| 44 | A0A6A5BXT8 | 2 | 44024 | Phosphoglycerate kinase |
| 43 | A0A6A5BJM7 | 2 | 28167 | Superoxide dismutase |
| 38 | A0A6A5BYE0 | 1 | 55166 | Cathepsin J |
| 59 | A0A6A5BEV3 | 1 | 32533 | Cathepsin X |
| 47 | A0A6A5CB00 | 1 | 79044 | Formyltetrahydrofolate synthetase |
| 49 | A0A1L1XWG8 | 1 | 38986 | Fowlerpain-3 |
| 32 | D2VQM7 | 1 | 37898 | GlcNAc kinase |
| 62 | A0A6A5BQM0 | 1 | 53450 | Isocitrate dehydrogenase [NADP] |
| 54 | A0A6A5BR74 | 1 | 35799 | Malate dehydrogenase |
| 52 | A0A6A5BSJ3 | 1 | 60876 | Peptidase S53 domain-containing protein |
| 53 | Q25558 | 1 | 13426 | Ubiquitin (Fragment) |

# Supplementary Table 4. Non redundant proteins in conditioned medium of *Naegleria fowleri*

# Limón

| **Protein Group** | **Accession** | **#Unique Peptide** | **Avg. Mass** | **Description** |
| --- | --- | --- | --- | --- |
| 1 | B5M6J9 | 5 | 41726 | Actin (Fragment) |
| 2 | A0A4V8H039 | 5 | 36595 | Glyceraldehyde-3-phosphate dehydrogenase |
| 5 | A0A6A5C7K8 | 4 | 59142 | CYTOSOL_AP domain-containing protein |
| 6 | A0A6A5BUH9 | 4 | 53729 | Dihydrolipoyl dehydrogenase |
| 4 | X5D911 | 3 | 34137 | Cathepsin B-like protein |
| 3 | A0A1L1XWF9 | 2 | 33944 | Fowlerpain-2 |
| 8 | A0A6A5BF81 | 2 | 38294 | Fructose-bisphosphate aldolase |
| 11 | A0A6A5C8E9 | 2 | 65714 | Guanine nucleotide-binding protein subunit beta-like protein |
| 7 | A0A6A5CFU9 | 2 | 42776 | Peptidase A1 domain-containing protein |
| 21 | D2VCL8 | 1 | 57680 | Actin bundling protein |
| 12 | D2UZU3 | 1 | 50952 | BPP domain-containing protein |
| 16 | X5D761 | 1 | 38387 | Cathepsin B protein |
| 15 | A0A6A5CAR5 | 1 | 48776 | Elongation factor 1-alpha |
| 18 | A0A1L1XWG7 | 1 | 38958 | Fowlerpain-3 |
| 14 | A0A6A5C6Q4 | 1 | 64041 | Lipase_3 domain-containing protein |
| 17 | D2V1F4 | 1 | 57577 | Methylmalonate-semialdehyde dehydrogenase (CoA acylating) |
| 20 | D2VTM5 | 1 | 84100 | Predicted protein |
